# Calcium Hydroxyapatite for Gingival Phenotype Enhancement: A Translational Review and Clinical Decision-Making Framework

**DOI:** 10.7759/cureus.112722

**Published:** 2026-07-15

**Authors:** Elsa Boustany

**Affiliations:** 1 Periodontology, Independent Research, Beirut, LBN

**Keywords:** aesthetics, calcium hydroxyapatite, dermal fillers, gingival recessions, interdental papilla

## Abstract

Soft-tissue deficiencies, including interdental papilla loss and thin gingival phenotypes, remain challenging to manage predictably in periodontology. While hyaluronic acid fillers provide transient volumetric improvement, calcium hydroxyapatite (CaHA) is a biostimulatory injectable biomaterial capable of inducing extracellular matrix remodeling and sustained soft-tissue reinforcement. This review evaluates the biological rationale and available evidence supporting CaHA and explores its potential role in periodontal soft-tissue management. A narrative review was conducted in accordance with the Scale for the Assessment of Narrative Review Articles (SANRA) recommendations. A structured search of PubMed, Scopus, and Google Scholar identified studies published from 2020 to 2025 investigating injectable CaHA in soft-tissue applications. Evidence from dermatology, aesthetic medicine, biomaterials, and histological research was synthesized to assess the biological mechanisms, tissue responses, and translational relevance of CaHA to gingival tissues. CaHA demonstrates a dual mechanism combining immediate volumization with delayed biostimulation through fibroblast activation, neocollagenesis, and extracellular matrix remodeling. Evidence from extraoral applications consistently reports increased tissue thickness, improved mechanical properties, and sustained clinical effects. Preliminary reports in periodontal contexts suggest potential for interdental papilla augmentation in mild defects; however, available data remain limited by small sample sizes, short follow-up, and lack of standardized protocols or histological validation. Current evidence supports the biological plausibility of CaHA as an adjunctive approach for soft-tissue reinforcement rather than defect correction. Its potential role appears most relevant in thin gingival phenotypes and mild papillary deficiencies, where increased tissue thickness and stability are desired. However, the available evidence is derived predominantly from non-periodontal studies, and the absence of controlled periodontal investigations precludes routine clinical use. Accordingly, the application of CaHA in periodontology should currently be regarded as experimental until validated by dedicated preclinical and clinical studies.

## Introduction and background

Regenerative periodontal procedures, including soft-tissue grafts, collagen matrices, and guided tissue regeneration, play a central role in the management of periodontally compromised teeth, yet their clinical outcomes can vary [1]. Soft-tissue deficiencies, particularly interdental papilla loss and mild gingival recessions, remain challenging to address [2]. Minimally invasive approaches such as gingival fillers have emerged as a potential strategy to increase soft-tissue volume. Hyaluronic acid (HA)-based fillers are the most studied and have demonstrated favorable outcomes in soft-tissue augmentation. In periodontology, they have been used in selected clinical settings to manage minor black triangles and thin gingival phenotypes. These fillers primarily provide temporary volume augmentation, and their effects diminish as the material is metabolized [3]. The limited durability of HA has prompted interest in alternative biomaterials capable of promoting structural tissue remodeling in addition to immediate volumization.

Calcium hydroxyapatite (CaHA)-based fillers, also known as Radiesse, are injectable biomaterials widely used in dermatology and aesthetic medicine for soft-tissue augmentation and biostimulation. In these fields, CaHA has demonstrated the capacity to increase soft-tissue volume, promote neocollagenesis, and support long-term tissue reinforcement [4]. Unlike HA, whose effects are predominantly volumetric, the biological properties of CaHA suggest the potential for longer-lasting extracellular matrix remodeling through collagen biostimulation. However, these findings originate predominantly from non-oral soft tissues and should not be directly extrapolated to periodontal tissues, which differ in their anatomical structure, vascularity, mechanical environment, and continuous exposure to the oral microbiome. Therefore, any potential periodontal application of CaHA should be regarded as a translational hypothesis rather than an established clinical indication, and its biological effects should not be interpreted as evidence of periodontal regeneration or new attachment formation.

Given the absence of periodontal clinical trials evaluating injectable CaHA, the evidence discussed in this review is derived predominantly from dermatology, aesthetic medicine, and biomaterials research. This narrative review aims to summarize the physicochemical properties, biological mechanisms, and clinical evidence of CaHa-derived primarily from non-periodontal literature. It further explores the biological rationale and potential translational relevance of CaHA in periodontology, including papilla reconstruction, management of mild gingival recession, and soft-tissue phenotype enhancement, while emphasizing that these proposed applications remain hypothetical and require validation through dedicated preclinical and clinical periodontal studies. Particular consideration is also given to the anatomical and biological characteristics of gingival tissues, including their limited tissue compliance and inflammatory environment, as well as the important distinction between soft-tissue augmentation and true periodontal regeneration.

## Review

Methodology

Study Design

This work was conducted as a narrative review in accordance with the Scale for the Assessment of Narrative Review Articles (SANRA) recommendations, aiming to synthesize existing scientific knowledge on CaHA used in soft-tissue augmentation and to explore its potential translational applications in periodontology [5]. Given the absence of periodontal studies specifically evaluating injectable CaHA, the review followed a qualitative, hypothesis-driven, and thematic approach, focusing on biological plausibility and interdisciplinary evidence. No systematic search protocol, meta-analysis, or quantitative data pooling was performed, in line with the narrative design of the review. As with all narrative reviews, this methodology is inherently more susceptible to author selection bias than a systematic review. Nevertheless, a structured search strategy and predefined eligibility criteria were applied to enhance methodological transparency and consistency.

Database Search Strategy

A non-systematic but structured literature search was conducted across the following databases: MEDLINE/PubMed, Scopus, and Google Scholar. These databases were selected to ensure comprehensive coverage of biomedical, clinical, and interdisciplinary literature, combining controlled indexing (PubMed), broad citation-based indexing (Scopus), and supplementary retrieval of grey and recently published literature (Google Scholar), which was considered appropriate given the exploratory nature of this narrative review. The literature search was conducted on December 19, 2025.

Keywords and Medical Subject Headings (MeSH) terms included “calcium hydroxyapatite,” “CaHA,” “dermal fillers,” “soft tissue augmentation,” and “collagen biostimulation.” The detailed search strategies for each database are presented below.

PubMed: (“calcium hydroxyapatite”[Title/Abstract] OR CaHA[Title/Abstract]) AND (“dermal fillers”[Title/Abstract] OR “soft tissue augmentation”[Title/Abstract] OR “collagen biostimulation”[Title/Abstract]).

Scopus: TITLE-ABS-KEY(“calcium hydroxyapatite” OR CaHA) AND TITLE-ABS-KEY(“dermal filler” OR “soft tissue augmentation” OR collagen biostimulation).

Google Scholar (Advanced Search): Exact phrase: “calcium hydroxyapatite.” At least one of the words: “dermal fillers”; “soft tissue augmentation”; “collagen biostimulation”.

Publication years: 2020-2025.

In PubMed, keywords lacking corresponding MeSH terms were entered in the Title/Abstract field, allowing the search to capture articles in which these terms appeared directly in the title or abstract. As Scopus and Google Scholar do not use MeSH indexing, the same keywords were applied using their native keyword-based search systems. Boolean operators (AND, OR) were used in PubMed and Scopus to combine or refine search concepts, whereas Google Scholar searches were conducted using the Advanced Search interface with equivalent keyword combinations and publication date filters.

Keywords related to periodontal tissues, such as “gingiva” and “interdental papilla,” were initially explored during preliminary searches. However, because these searches yielded very limited literature directly evaluating injectable CaHA in periodontal applications, the scope of the review was intentionally expanded to include non-periodontal soft-tissue studies to explore the biological rationale and potential translational relevance of CaHA in periodontology, rather than to exclude periodontal evidence.

Titles and abstracts retrieved from each database were initially screened for relevance, followed by full-text assessment according to the predefined eligibility criteria.

Only articles published between 2020 and 2025 were considered eligible and were retrieved by applying date restrictions directly within each database. This timeframe was selected to emphasize contemporary evidence regarding injectable calcium hydroxyapatite and its emerging clinical applications. Nevertheless, earlier landmark publications were consulted where necessary to provide biological context and facilitate interpretation of the underlying biological mechanisms. Additional literature on skin histology, gingival histology, and alternative soft-tissue fillers was consulted to contextualize and interpret the findings, but was not included as part of the primary literature search results.

Inclusion and Exclusion Criteria

Articles were included if they investigated injectable CaHa as a soft-tissue biomaterial, provided original research data or synthesized scientific evidence (including clinical studies, in vivo and in vitro experiments, histological analyses, or systematic reviews and meta-analyses), and were relevant to dermatology, aesthetic medicine, or biomaterials. Additional inclusion criteria required that studies report mechanisms relevant to soft-tissue biology, such as collagen synthesis, fibroblast activation, or extracellular matrix remodeling. Animal studies were also included when they contributed to the mechanistic understanding of CaHA-induced tissue responses.

Articles were excluded if they consisted solely of opinion pieces or commentary without supporting scientific data, or if CaHA was combined with unrelated compounds that prevented isolated evaluation of its specific effects.

Results

**Figure 1:**
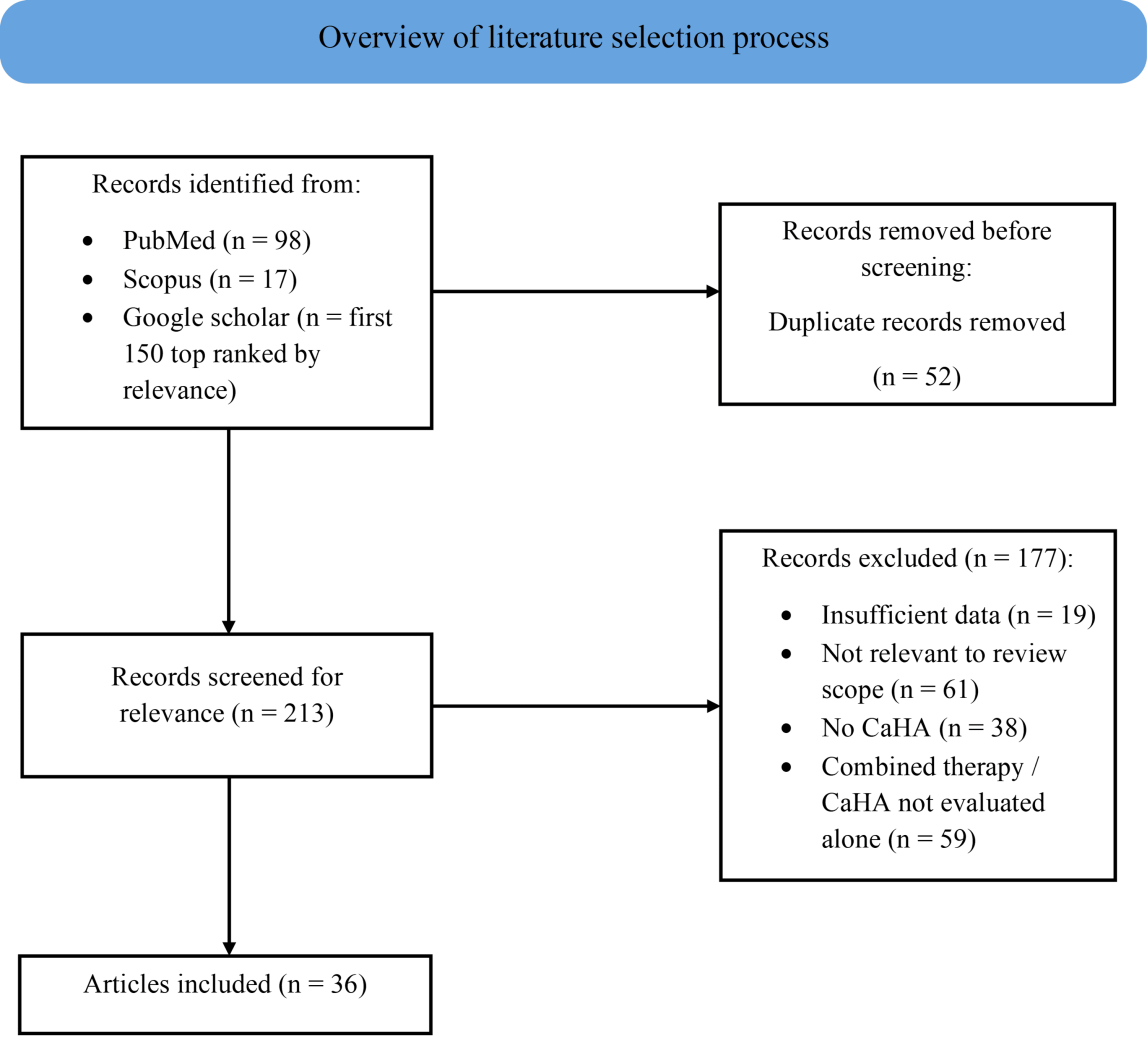
Overview of the literature selection process.

Articles published before 2020 were automatically excluded from the initial results. Duplicates were then removed, and study selection was performed in two consecutive screening phases: an initial screening based on titles and abstracts, followed by full-text assessment of potentially relevant articles. For Google Scholar, only the first 150 results ranked by relevance were screened, in accordance with the platform’s retrieval approach. For clarity, the title/abstract screening and full-text eligibility assessment are presented as a single screening stage in Figure 1. Following the selection process, 36 studies met the predefined eligibility criteria and were included in the narrative review (Figure 1).

Discussion

While the included studies suggest a favorable tissue response to CaHa, interpretation of these findings requires consideration of biological mechanisms and study limitations. Therefore, the discussion focuses on the histological basis of CaHA-induced biostimulation, its inflammatory profile, and the applicability of existing evidence to periodontal soft tissues.

Physicochemical Characteristics of Calcium Hydroxyapatite

CaHa fillers consist of biodegradable CaHA microspheres suspended in a carboxymethylcellulose (CMC) gel carrier. In the commercially available formulation Radiesse®, CaHA microspheres with a diameter of approximately 25-45 μm are dispersed within an aqueous gel composed primarily of CMC, glycerin, and water. The gel carrier accounts for approximately 70% of the total volume, while CaHA microspheres constitute about 30% [6-9]. CaHa is a naturally occurring mineral and represents the principal inorganic component of human bone and teeth, a property that contributes to its recognized biocompatibility. Its particles are broken down through normal physiological processes, making it non-toxic, non-mutagenic, non-antigenic, non-allergenic, and safe for systemic and local use [10,11].

Biological Effects of Calcium Hydroxyapatite on Soft-Tissue Remodeling

CaHa combined with CMC exhibits a two-fold mechanism of action. Initially, the CMC gel serves as a carrier, evenly distributing CaHA microspheres at the injection site. It provides immediate volumization and temporary tissue support while gradually degrading over two to four months. The particulate structure of CaHA microspheres acts as a temporary scaffold that supports fibroblast activity. In addition, CaHA has been reported to promote fibroblast activity through mechanotransduction, which contributes to increased deposition of collagen types I and III, elastin, and proteoglycans, as well as angiogenic responses, thereby supporting extracellular matrix remodeling [7,9,12-16]. Through multiple complementary pathways, CaHA microspheres also stimulate biostimulation by restoring extracellular matrix tension and counteracting matrix metalloproteinases-mediated collagen degradation. In addition, CaHA may promote the deposition of other extracellular matrix components, including proteoglycans, which contribute to connective tissue organization, hydration, and mechanical stability [17]. CaHA microspheres can persist at the injection site for 12-24 months before degradation, while the volumizing effects of CaHA-CMC have been observed to last up to 36 months, highlighting its capacity for both immediate and long-term tissue enhancement. Furthermore, CaHA is characterized by high viscoelasticity, which allows it to remain localized at the injection site without migrating [8,18,19].

When CaHA-CMC is injected, the host response is influenced by particle size. Particles smaller than 10 μm are typically engulfed by single macrophages, whereas particles larger than 10 μm induce the formation of multinucleated giant cells (foreign body giant cells, FBGCs), which mediate their gradual degradation over time. Given that CaHA microspheres in commercially available formulations, such as Radiesse®, range from approximately 25-45 μm in diameter, degradation is expected to predominantly involve FBGC-mediated processes rather than single-cell phagocytosis. Histological observations indicate that this process occurs without chronic inflammation or granuloma formation, suggesting a controlled and self-limited foreign body reaction. However, the precise molecular mechanisms underlying FBGC formation and CaHA degradation remain incompletely understood [19].

Immunologic and Inflammatory Profile of Calcium Hydroxyapatite

The immunologic and inflammatory profile of injectable biomaterials is a critical determinant of their biocompatibility, particularly in immune-active tissues such as the oral mucosa and gingiva. For CaHa, understanding host immune interactions is essential to evaluate its suitability for soft-tissue applications.

Macrophage responses play a central role in biomaterial-induced soft tissue remodeling. In the context of injectable biomaterials, including CaHa, implantation has been associated with a shift in macrophage polarization from a predominantly pro-inflammatory M1 phenotype toward an anti-inflammatory and pro-regenerative M2 phenotype as part of the foreign body response. M2 macrophages are characterized by the secretion of anti-inflammatory mediators, including interleukin-10 and transforming growth factor-β, which contribute to the resolution of inflammation and support fibroblast recruitment and activation [20,21]. These fibroblasts can further differentiate into myofibroblasts, promoting extracellular matrix remodeling through collagen synthesis and tissue reorganization. In addition, M2 macrophages exhibit pro-angiogenic activity, partly mediated through vascular endothelial growth factor signaling, thereby contributing to neovascularization and tissue repair [19].

Differences in particle morphology have been proposed to influence host immune responses to injectable biomaterials. In this context, the relatively uniform and spherical morphology of CaHa microspheres has been associated with reduced phagocytosis and limited inflammatory cell recruitment in histological observations. By contrast, more irregular particle geometries have been suggested to promote sustained macrophage activation and a more pronounced inflammatory response [7]. Collectively, these observations suggest that the foreign body response to CaHA is generally controlled and self-limited, allowing progressive tissue remodeling while avoiding persistent inflammatory activation following implantation [22].

Such a controlled foreign body response is considered favorable for biomaterial integration because it is associated with macrophage phenotypes that support tissue remodeling rather than chronic inflammatory reactions. The available preclinical and histological evidence suggests that CaHA is generally associated with a controlled inflammatory response and favorable tissue integration in non-oral soft tissues. However, these findings should not be interpreted as direct evidence of comparable biological behavior in gingival tissues, where differences in anatomy, immune activity, and continuous microbial exposure may influence host responses. Consequently, extrapolation of these immunologic observations to periodontal applications should be regarded as biologically plausible but remains unvalidated in dedicated periodontal studies.

Clinical and Histological Evidence of Calcium Hydroxyapatite in Soft Tissue

Aesthetic soft-tissue applications represent the largest body of clinical and histological evidence available for CaHa in soft tissues. Although not specific to the oral environment, these studies provide relevant insights into tissue integration, remodeling, durability, and safety. However, because these findings originate predominantly from dermatologic and facial soft tissues, their applicability to periodontal tissues should be interpreted with caution. Differences in tissue architecture, vascularity, immune environment, and continuous microbial exposure may influence both tissue responses and clinical outcomes.

Clinical investigations have demonstrated that hyperdiluted CaHA can induce progressive soft-tissue volume enhancement over time, reflecting collagen-driven tissue remodeling rather than simple filler persistence [6,23]. CaHA supports fibroblast activation and the synthesis of new extracellular matrix components, including collagen and elastin [17]. Comparative studies further indicate that CaHA provides greater mechanical support and lifting capacity than HA fillers, while maintaining positional stability without excessive volumization. Imaging studies and long-term follow-up data suggest that these effects persist over extended periods and are primarily attributed to CaHA-induced collagen deposition rather than the continued physical presence of the filler material itself [12,24,25]. In addition, investigations employing customized CaHA dilution protocols have reported sustained clinical improvements lasting up to 12 to 30 months, supporting the durability and favorable safety profile of CaHA as a long-term biostimulatory treatment in dermatologic applications [26-28].

Beyond volumetric correction, CaHA has been shown to enhance skin quality through fibroblast-mediated stimulation of collagen types I and III, elastin, and proteoglycans. This regenerative activity complements the initial mechanical support provided by CaHA filler, resulting in sustained improvements in dermal thickness, elasticity, and overall mechanical properties. Some studies reported increased collagen deposition that persisted for up to 78 weeks following injection. Objective assessments further demonstrate improved skin hydration, reduced roughness, and neovascularization, supporting CaHA’s distinct profile as a biostimulatory filler compared with HA, poly-L-lactic acid, or polycaprolactone-based materials [29-32]. Although these findings provide a valuable biological framework for understanding the tissue response to CaHA, they should be regarded as indirect evidence when considering potential periodontal applications. Dedicated preclinical and clinical studies in gingival tissues remain necessary before comparable biological behavior or clinical efficacy can be assumed.

Potential Applications of Calcium Hydroxyapatite in Periodontology

The gingiva is a specialized component of the oral mucosa characterized by a dense connective tissue covered by a keratinized stratified squamous epithelium and firmly anchored to the underlying alveolar bone and cementum. Its structural integrity depends on a highly organized extracellular matrix rich in collagen fibers, elastin fibers, glycoproteins, and proteoglycan polysaccharide complexes. Gingival connective tissue papillae interdigitate with epithelial rete pegs, contributing to mechanical stability and resistance to functional stress. In addition to fibroblasts, the gingival tissue contains immune-active cell populations, reflecting its constant exposure to microbial and mechanical challenges. Homeostasis of the gingiva relies on tightly regulated fibroblast activity and preservation of collagen architecture, which are essential for maintaining gingival firmness, contour, and resistance to recession [33].

**Figure 2:**
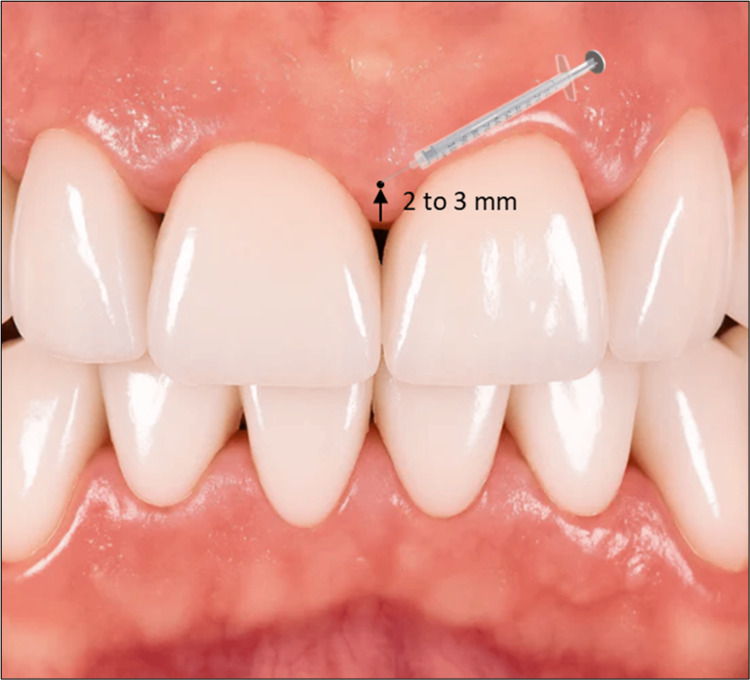
Schematic representation of the interdental injection technique with one injection point. The author confirms that this figure was created using original illustrations and no artificial intelligence-generated images were used.

Interdental papilla volume deficiency: Although evidence in periodontology remains scarce, limited clinical reports have explored the use of CaHA for the treatment of papillary deficiencies in the esthetic zone to minimize black triangles. A clinical study investigated the use of CaHa for minimally invasive augmentation of deficient interdental papillae in periodontally healthy patients with intact interdental bone architecture. CaHA was injected in small volumes (approximately 0.2 mL) using a fine-gauge needle, with the material deposited 2 to 3 mm apical to the coronal tip of the papilla under local anesthesia. In some cases, CaHA was blended with lidocaine to facilitate injection and improve patient comfort. The interdental injection technique with a single injection point is illustrated schematically in Figure 2.

Clinical outcomes were evaluated using standardized clinical indices and objective morphometric analysis, including the Papilla Presence Index, Papillary Marginal Gingival Index, probing depth, clinical attachment level, and interdental papilla height measured on calibrated digital photographs with image-analysis software. Follow-up assessments up to six months demonstrated a significant and sustained increase in papillary height and embrasure fill, with improvements remaining stable and without adverse effects on periodontal parameters, supporting the feasibility and short-term safety of CaHA injections for interdental papilla volume reconstruction [34]. However, these findings should be interpreted cautiously because they originate from a limited number of clinical investigations with relatively short follow-up periods. Although papilla loss was classified using the Papilla Presence Index, treatment outcomes were not stratified according to baseline papilla severity.

**Figure 3:**
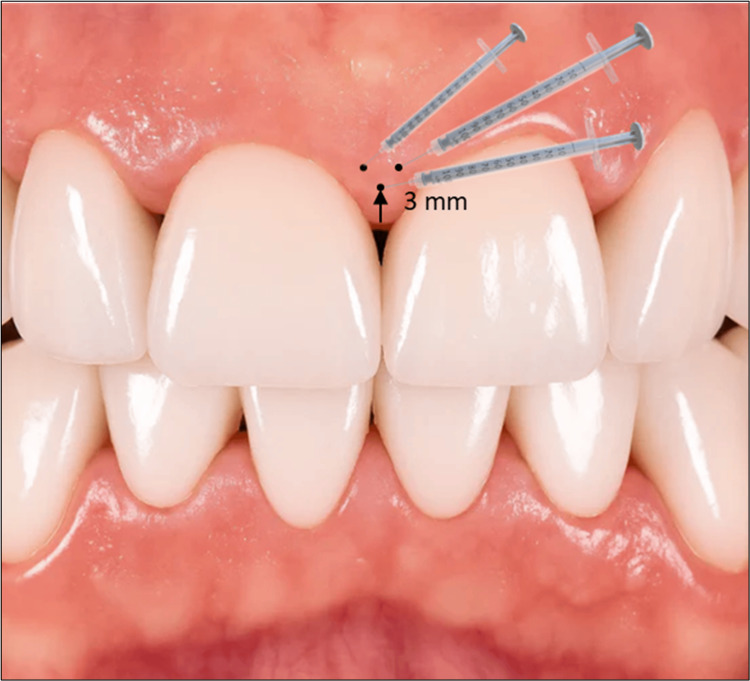
Schematic representation of the interdental injection technique with three injection points. The author confirms that this figure was created using original illustrations and no artificial intelligence-generated images were used.

Another study employed a hyperdiluted CaHa mixture (Radiesse®) with lidocaine and saline at a 1:2 ratio to reduce viscosity and improve distribution, which was then combined with diluted HA (Balance®) to create a single injectable “mesoblend.” The injection was performed using a micro-aliquot technique via a 30-gauge insulin syringe at three points per interdental papilla: 3 mm from the papillary tip, at the mesial line angle, and at the distal line angle of the adjacent teeth, delivering a total volume of 0.1 mL per site, followed by gentle massage to ensure even distribution. The interdental injection technique with three injection points is illustrated schematically in Figure 3.

Outcomes were assessed both objectively, via interdental papillary height measured with a calibrated periodontal probe and Papillary Fill Index, and subjectively, through patient satisfaction scores and visual analog scales for aesthetics. The results demonstrated a significant increase in papillary height and Papillary Fill Index as early as two weeks post-injection, with improvements maintained up to six months and minimal regression. Patient-reported outcomes were consistent with objective measures, showing high satisfaction and aesthetic improvement. The proposed mechanism of action is thought to involve CaHA microspheres providing immediate volumization and long-term biostimulation by acting as scaffolds that stimulate fibroblast activity, collagen deposition, and extracellular matrix formation, resulting in more durable tissue augmentation compared to HA alone [35].

These findings highlight the need for a unified papilla defect classification, such as the Nordland & Tarnow classification (1998), to be applied consistently across studies to standardize reporting and clarify whether outcomes are generalizable or restricted to mild papilla deficiencies [36]. In addition, current evidence remains limited by short follow-up periods, small sample sizes, and a lack of histological validation in gingival tissues. These findings should therefore be interpreted cautiously, emphasizing the need for larger, controlled, and histologically informed studies to confirm the clinical efficacy and long-term safety of CaHA injections in gingival tissues.

**Figure 4:**
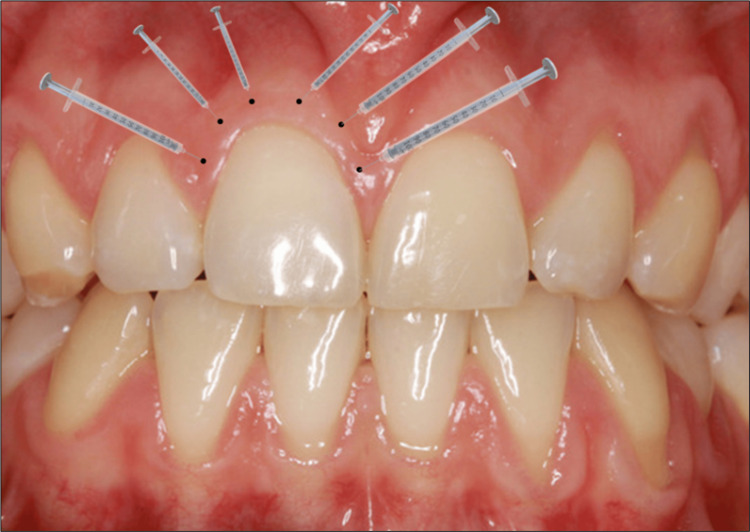
Schematic illustration of the proposed injection approach for gingival phenotype enhancement and recession management. The author confirms that this figure was created using original illustrations and no artificial intelligence-generated images were used.

Gingival phenotype modification: Beyond interdental papilla reconstruction, soft-tissue volumization concepts may be extended to gingival phenotype modification, particularly in sites characterized by a thin gingival biotype. Gingival thickness is a key determinant of tissue stability, as thicker tissues exhibit greater mechanical resistance and improved tolerance to functional and inflammatory challenges, whereas a thin gingival phenotype represents a risk factor for recession, rather than a direct triggering factor [37]. This relationship is further illustrated by age-related changes in the gingival structure. With aging, gingival recession becomes increasingly prevalent, a process associated with progressive collagen degradation, reduced fibroblast activity, and gradual loss of extracellular matrix organization over time [38]. The proposed injection strategy is schematically illustrated in Figure 4.

Injectable CaHa should not be considered a replacement for established mucogingival or root coverage procedures, but rather a potential adjunctive approach aimed at reinforcing gingival tissues in selected clinical situations. Through its immediate volumizing effect and subsequent biostimulatory activity, CaHA may enhance collagen density and extracellular matrix integrity, contributing to increased gingival thickness rather than coronal tissue displacement. This distinction is critical, as the therapeutic objective in phenotype modification is tissue reinforcement and stabilization rather than root coverage.

From a biological standpoint, the potential relevance of CaHA-induced tissue thickening may extend beyond purely mechanical effects. By promoting a reparative tissue environment, CaHA may favor macrophage polarization toward an anti-inflammatory, pro-regenerative (M2) phenotype, which is associated with collagen remodeling, angiogenesis, and extracellular matrix maturation. Such an immunomodulatory profile could support gingival tissue homeostasis and contribute to improved resilience against low-grade inflammation. Whether these biological effects translate into meaningful clinical benefits in periodontal tissues remains unknown and requires validation through dedicated preclinical and clinical studies.

Clinical Positioning and Decision-Making Framework

From a clinical standpoint, the potential application of CaHa should be restricted to clearly defined indications rather than generalized use. Given the heterogeneity of soft-tissue defects and the absence of standardized periodontal protocols, its use is best interpreted within a structured decision-making framework. Within this framework, CaHA appears most relevant in patients presenting with a thin gingival phenotype or mild interdental papilla deficiency, where the therapeutic objective is to enhance tissue thickness and mechanical stability rather than to achieve defect correction.

In contrast, there is currently no evidence supporting the use of CaHA in advanced gingival recession (RT2/RT3) or in moderate-to-severe papilla loss, where interproximal attachment loss is present. In these clinical scenarios, established surgical approaches, including connective tissue grafting and mucogingival procedures, remain the standard of care. This distinction is essential to avoid misinterpretation of CaHA as a substitute for regenerative or root coverage techniques. CaHA occupies an intermediate role between minimally invasive fillers, such as HA, and surgical interventions. Its minimally invasive nature and biostimulatory properties make it a potential option for early intervention and phenotype modification; however, its clinical use should remain selective and biologically guided until validated by controlled periodontal studies.

Limitations of the existing evidence

The current evidence supporting the potential use of injectable CaHa in periodontology remains limited and should be interpreted with caution. Most available data originate from dermatology and facial aesthetic medicine rather than oral or periodontal tissues. Consequently, the biological responses observed in skin cannot be assumed to accurately reflect those of the gingiva, which differs substantially in tissue architecture, vascularity, immune activity, biomechanical loading, and continuous exposure to the oral microbiome. In addition, the available studies are characterized by relatively small sample sizes, heterogeneous treatment protocols, variable follow-up periods, and differences in CaHA concentration, dilution, injection depth, and injected volume, limiting direct comparison between studies. Publication bias may also be present, as positive clinical outcomes are more likely to be reported than unfavorable or neutral findings. Collectively, these limitations restrict the strength of current evidence and highlight the need for cautious interpretation when considering potential periodontal applications.

Future perspectives

Despite its generally favorable safety profile, CaHa injections have been associated with early and delayed adverse reactions, including nodules, granulomatous inflammation, infections, and, more rarely, vascular complications leading to ischemia or tissue necrosis [13,39,40]. However, these adverse events have been predominantly reported in dermatologic and facial aesthetic applications, and their relevance to gingival tissues remains uncertain. Differences in vascularization, immune response, and tissue architecture between skin and gingiva may influence both the nature and frequency of such reactions.

In addition, variability in injection protocols, including product concentration, dilution ratios, depth of placement, and injected volume, represents a major source of heterogeneity, highlighting the need for systematic evaluation of CaHA dilution strategies to optimize both safety and biological outcomes [41]. A hybrid formulation combining CaHa with HA, such as HArmonyCa, may represent a potential strategy to balance immediate volumization with longer-term biostimulatory effects, although its clinical and biological advantages remain to be established [10].

Before clinical adoption in periodontology can be considered, dedicated preclinical and translational investigations are required. Gingival-specific animal models incorporating oral microbial exposure should evaluate local inflammatory responses, collagen organization, extracellular matrix remodeling, and microbiome interactions following CaHA injection. Histomorphometric studies are needed to characterize newly formed collagen, its orientation and maturation, and its relationship with adjacent periodontal structures, including cementum and the periodontal ligament, when CaHA is placed near the interdental papilla. Furthermore, dose optimization studies should investigate the influence of dilution protocols, injection depth, and injected volume using standardized clinical and blinded histological outcome measures. Long-term safety studies should also evaluate potential complications, including infection, delayed granuloma formation, effects on probing depth, and plaque retention.

Future clinical studies should incorporate standardized and clinically relevant outcome measures, including interdental papilla height and volume assessed by calibrated photography and three-dimensional imaging, gingival thickness measured by ultrasound or transgingival probing, probing depth, clinical attachment level, bleeding on probing, patient-reported esthetic outcomes, and long-term complication rates. The use of standardized defect classifications and injection protocols would further improve comparability across future investigations.

Overall, CaHa should not be viewed as an alternative to established periodontal therapies but rather as a potential adjunct for soft-tissue phenotype enhancement in carefully selected clinical situations. Although the available evidence and biological rationale suggest promising translational potential, its application in periodontology should currently be regarded as experimental until supported by well-designed preclinical studies and controlled clinical trials specifically conducted in periodontal tissues.

## Conclusions

CaHa is a biostimulatory injectable biomaterial supported by extensive clinical and histological evidence in extraoral soft tissues, where it has been shown to induce extracellular matrix remodeling, collagen synthesis, and durable tissue reinforcement. Its mechanism of action, combining transient volumization with longer-term biostimulation, distinguishes CaHA from purely volumetric fillers. However, the available evidence is derived predominantly from dermatologic and aesthetic medicine, and its direct applicability to periodontal tissues remains unproven. In the absence of dedicated periodontal clinical trials, any consideration of CaHA within periodontal soft tissues must remain theoretical and based on biological plausibility rather than demonstrated clinical efficacy. Extrapolation from dermatologic and aesthetic medicine suggests that CaHA could be conceptually explored as an adjunctive approach for soft-tissue reinforcement in specific periodontal contexts, such as interdental papilla volume deficiency or thin gingival phenotypes. Importantly, such an approach would aim at enhancing tissue thickness and resilience rather than correcting existing defects or achieving root coverage. Future research should prioritize gingival-specific preclinical models and controlled clinical studies with standardized defect classification, optimized dilution and injection protocols, and histological evaluation to clarify tissue responses, inflammatory behavior, and long-term safety within gingival tissues. Standardized clinical outcome measures, including gingival thickness, interdental papilla dimensions, periodontal parameters, patient-reported esthetic outcomes, and long-term complication rates, will also be essential to facilitate comparison across future studies. Until such data are available, CaHA should be regarded as an experimental and biologically plausible, yet currently unvalidated, adjunctive approach in periodontal soft-tissue management.
